# Can coffee silverskin be a useful tool to fight metabolic syndrome?

**DOI:** 10.3389/fnut.2022.966734

**Published:** 2022-09-21

**Authors:** Nelson Andrade, Juliana A. Barreto Peixoto, M. Beatriz P. P. Oliveira, Fátima Martel, Rita C. Alves

**Affiliations:** ^1^REQUIMTE/LAQV, Department of Chemical Sciences, Faculty of Pharmacy, University of Porto, Porto, Portugal; ^2^Unit of Biochemistry, Department of Biomedicine, Faculty of Medicine of Porto, University of Porto, Porto, Portugal; ^3^Instituto de Investigação e Inovação em Saúde (I3S), University of Porto, Porto, Portugal

**Keywords:** coffee by-products, caffeine, chlorogenic acids, melanoidins, metabolic syndrome

## Abstract

Coffee is one of the most consumed products in the world, and its by-products are mainly discarded as waste. In order to solve this problem and in the context of a sustainable industrial attitude, coffee by-products have been studied concerning their chemical and nutritional features for a potential application in foodstuffs or dietary supplements. Under this perspective, coffee silverskin, the main by-product of coffee roasting, stands out as a noteworthy source of nutrients and remarkable bioactive compounds, such as chlorogenic acids, caffeine, and melanoidins, among others. Such compounds have been demonstrating beneficial health properties in the context of metabolic disorders. This mini-review compiles and discusses the potential health benefits of coffee silverskin and its main bioactive components on metabolic syndrome, highlighting the main biochemical mechanisms involved, namely their effects upon intestinal sugar uptake, glucose and lipids metabolism, oxidative stress, and gut microbiota. Even though additional research on this coffee by-product is needed, silverskin can be highlighted as an interesting source of compounds that could be used in the prevention or co-treatment of metabolic syndrome. Simultaneously, the valorization of this by-product also responds to the sustainability and circular economy needs of the coffee chain.

## Introduction

Coffee is one of the most produced, traded, and consumed commodities worldwide. Only in 2020/2021, around 10.13 and 10.06 million tons of coffee beans were produced and consumed, respectively, around the globe (1). Despite the multiplicity of existing and identified coffee species, *Coffea arabica* L. and *Coffea canephora* Pierre (known as arabica and robusta, respectively) are the most important in the international coffee trade, representing around 98% of the market (2). Besides the unique sensory and pleasant flavor of coffee, which alone contribute significantly to the popularity and attractiveness of this beverage, its regular consumption has been associated to several health benefits (3). The functional activity of coffee is attributed to a wide range of bioactive components such as phenolic compounds (mainly chlorogenic acids), alkaloids (caffeine and trigonelline), diterpenes (cafestol and kahweol), and other secondary metabolites (4). Improvements in mental alertness (5), reduced risk of diseases development [type 2 diabetes (6), depression (7), suicidal behavior (8), cancer (9), hepatic injuries (10, 11), neurodegenerative diseases (12), and cardiovascular disorders (13)], and positive effects on the gastrointestinal tract and gut microbiota (14) are well–documented benefits.

On the other hand, coffee processing involves different serial operations which generate distinct by-products (husks, pulp, mucilage, parchment, silverskin, and spent coffee grounds) (15). In total, coffee industries produce more than 10 million tons of solid residues per year (16), representing a serious environmental problem. Basically, all of them hold a significant nutritional and bioactive potential, therefore, their use/recycling into health promoting products is of great interest and can also bring positive socioeconomic and environmental impacts (17, 18).

Coffee silverskin, in particular, is a thin tegument that covers the green coffee beans and is detached during the roast when these expand, being the major by-product of coffee roasting companies (19). Although it is generally discarded, silverskin has also been highlighted as a natural source of dietary fiber, protein and bioactive compounds that can be further used, directly of after extraction, in the development of several functional food products (20, 21).

Metabolic syndrome (MetS) is a set of metabolic disorders (e.g., insulin resistance, hypertriglyceridemia, and abdominal obesity) that increases the risk for type 2 diabetes, cardiovascular diseases, and even cancer (22). A sedentary lifestyle, unhealthy eating habits (e.g., high sugar consumption), and increased stress, are factors that play a crucial role in this syndrome (23). Type II diabetes and obesity emerged as major causes of MetS, which is significantly increasing in modern societies (24). According to The International Diabetes Federation, one-quarter of the world's population has MetS (25). This alarming estimation highlights the urgent need for new ways of preventing and/or treating this syndrome.

Plant extracts have been attracting much attention as potential preventive or co-therapeutic agents against MetS due to their multiple targets and lower side effects (26). In this context, much attention has been given to natural bioactive compounds, namely those which can be found in coffee silverskin, as they have been presented as a weapon for the prevention and treatment of MetS (27, 28). The aim of this mini review is to explore the current evidence linking coffee silverskin and its main bioactive compounds to a lower risk of developing MetS, emphasizing the known mechanisms of action that could be involved in such protection.

## Nutritional and chemical composition of coffee silverskin

**Figure 1:**
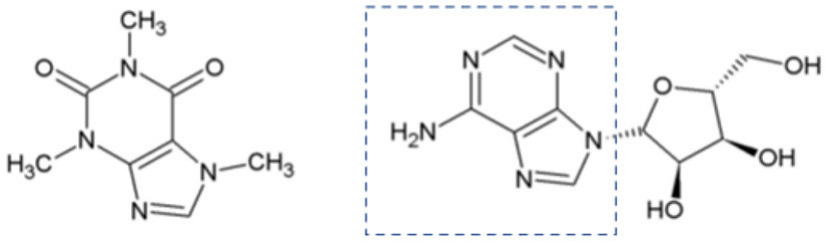
Chemical structures of caffeine (left) and adenosine (right).

Note: Adenosine is a potent endogenous neuromodulator, which inhibits the release of several neurotransmitters, such as glutamate, gamma-aminobutyric acid, acetylcholine and monoamines (33).

Silverskin is rich in dietary fiber (~60 g/100 g, mainly insoluble one) (29) and protein (~12 g/100 g) (30), but its fat content is low (often below 3g/100 g) (29, 31). It is also a source of minerals (8 g/100 g in total), mainly potassium (5 g/100 g), magnesium (2 g/100 g), and calcium (0.5 g/100 g) (29). The principal amino acids found in silverskin protein are aspartic acid (10.2 mg/g) and glutamic acid (9.2 mg/g), however branched chain amino acids (leucine, isoleucine, and valine) are also present in significant levels (7.6, 4.7, and 5.0 mg/g) (30). Moreover, hydrosoluble vitamins B_2_ (0.18–0.2 μg/g), and B_3_ (2.5–3.1 μg/g) (32) are also present, as well as the liposoluble vitamin E, which is represented by different vitamers (being α-tocopherol the major one: 22.5 μg/g) (29). Silverskin also contains caffeine (~1 g/100 g), a widely recognized coffee stimulant that acts mainly by inhibiting adenosine receptors (33). In Figure 1, the structural similarity between caffeine and adenosine is highlighted. Other action mechanisms of caffeine are also described, such as inhibition of phosphodiesterases (cyclic AMP inactivating enzymes) or mobilization of intracellular calcium (33).

**Figure 2:**
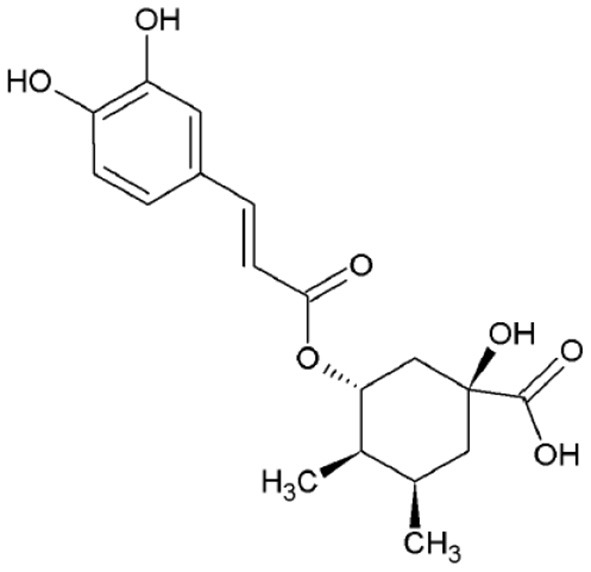
Chemical structure of 5-caffeoylquinic acid.

As for coffee beans, chlorogenic acids (GCA) are the major phenolics present in silverskin. CGA represent a whole set of hydroxycinnamic esters with quinic acid. It is known that this group of compounds plays crucial roles on the regulation of glucose and lipid metabolism, with several beneficial health effects described (e.g., antidiabetic, anti-inflammatory, antitumoral, etc.,) (34). The major chlorogenic acid of silverskin is 5-caffeoylquinic acid (0.1–0.2 g/100 g; Figure 2), but other CGA are also present, namely, 3-caffeoylquinic acid, 4-caffeoylquinic acid, 3-feruloylquinic acid, 5-feruloylquinic acid, and dicaffeoylquinic acids, among others (35).

In addition to these bioactive components, melanoidins are also formed during coffee roasting. These are a complex group of polymeric structures generated during the last stage of the Maillard reaction, being responsible for the brown color, taste, and texture of foods exposed to high temperatures (36). Melanoidins result from reactions among the amino group of amino acids, proteins, or vitamins and the carbonyl group of reductive sugars or oxidized lipids. Their chemical structure comprises, thus, nitrogen-containing anionic compounds of high molecular weight (37). Phenolic compounds can also be linked to the melanoidin nucleus *via* covalent interactions or through non-covalent bonds, being released during intestinal digestion (36, 38). Indeed, it has been reported that some melanoidins are extensively metabolized by gut microbiota also leading to the production of short chain fatty acids and favoring the growth of beneficial bacteria (39).

## Effects of the main bioactive components of coffee silverskin on metabolic syndrome

The main bioactive compounds present in coffee silverskin (above detailed) can affect several pathways involved in the pathogenesis of MetS. In fact, they have been associated to beneficial effects on type II diabetes (40, 41), obesity (42), insulin resistance (43), oxidative stress (40, 44), regulation of glycemic and lipid disorders (45), and other MetS features. Those aspects and mechanisms will be presented and discussed below.

It is also important to briefly highlight the role of metabolites, although they are not the main focus of this mini review. For example, extensive research on CGA metabolism has revealed that one-third of these consumed compounds are absorbed throughout the digestive tract and further metabolized (e.g., by partial intestinal hydrolysis, phase two metabolism involving sulfation and glucuronidation…), with a wide range of variability among individuals. According to a recent study, for instance, ferulic acid undergoes extensive phase II metabolism in the liver due to the presence of hydroxyl groups (conjugation reactions—glucuronic acid and ferulic acid sulfate are the major metabolites in rat plasma and urine) (46). In turn, the unabsorbed CGA are extensively hydrolyzed by gut bacteria, resulting in phenolic acids which are then methylated, conjugated with sulfate or glycine (mostly CGA colonic metabolites: ferulic, isoferulic, and hydroxybenzoic acids), functioning as a potential prebiotic for beneficial bacteria (47, 48).

Regarding caffeine (whose biological effects are mostly dependent on its biotransformation in the body), as it is extensively metabolized in the liver by phase I (cytochrome P450) enzymes, paraxanthine, xanthines, and methyluric acids are the main metabolites found in plasma and urine (49).

Further, melanoidin's carbohydrates are typically metabolized inside the colon by gut microbiota to short-chain fatty acids (acetate, lactate, butyrate, and propionate) having a important role in gut homoeostasis, digestion, and illness prevention.

Nevertheless, because there is still some debate regarding how these metabolites are assessed (methodologically and in terms of the models utilized), more research on their bioavailability, concentrations, and activity in different tissues and organs after absorption is required.

### I—Effect on intestinal sugar absorption

**Figure 3:**
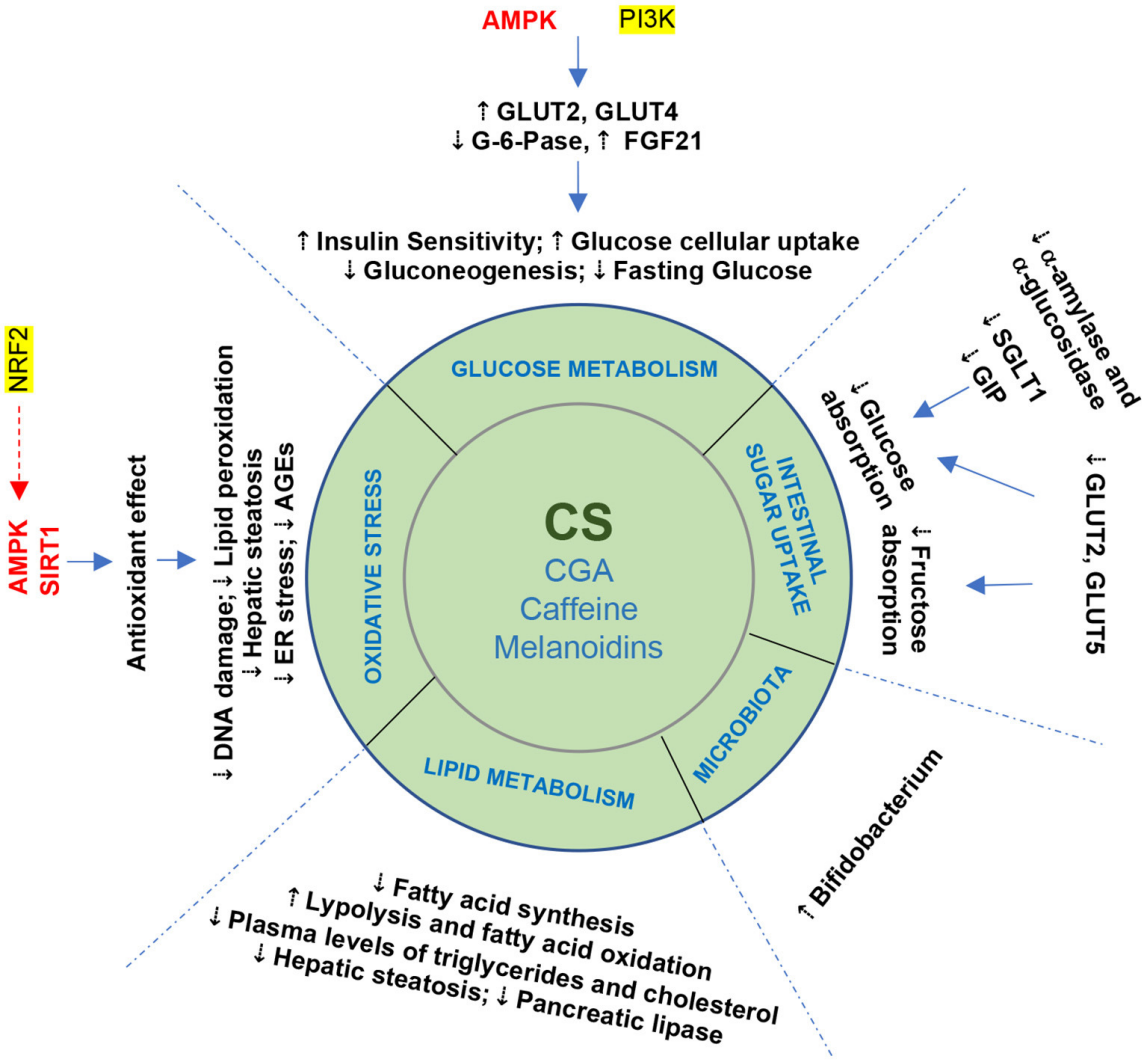
Beneficial effects of coffee silverskin and its bioactive compounds on metabolic syndrome: summary of the potential mechanisms involved.

Regarding the effect of silverskin components on glucose uptake, CGA have been found to reduce the intestinal absorption of glucose in rats, by stimulating dispersal of the Na^+^ electrochemical gradient, thus inhibiting Na^+^-dependent glucose absorption (45, 50). In fact, direct effects on glucose tolerance appear to be caused by the antagonistic effect of CGA (with/without caffeine) on glucose transport, shifting glucose absorption to more distal parts of the intestine (45). CGA (mainly 5- and 3-CQA) also affects glucose absorption through the reduction of α-amylase and α-glucosidase activities and α-amylase activity is also decreased in the presence of caffeine, resulting in reduced uptake of glucose (51) and suggesting a positive role in glycaemic control (52) (Figure 3), Further, CGA from coffee sources (coffee silverskin included) was able to significantly attenuate the postprandial release of glucose-dependent insulinotropic polypeptide (GIP), a gut hormone that potentiates insulin secretion, in the proximal part of the small intestine (53). As the amount of glucose absorbed at the intestinal barrier determines the magnitude of the GIP response, these results suggest that CGA (5-CQA) is likely to decrease the intestinal absorption of glucose (34). Additionally, melanoidin metabolites stimulate the secretion of GLP-1 by the enteroendocrine cells, causing a delay in the digestion of sugars (41).

*In vitro*, a subset of coffee CGAs reduced glucose absorption in intestinal Caco-2 cells (54). Further, among the potential bioactive-derived metabolites present in the coffee silverskin, ferulic acid has shown effects on glucose uptake. In fact, another *in vitro* study in adipocyte cells suggested that ferulic acid enhances glucose uptake through the PI3K-dependent pathway (55).

Moreover, a recent work suggested that coffee silverskin aqueous extract is also rich in other phenolics, such as phenolic acids (caffeic, ferulic, gallic, *p*-coumaric, syringic, and vanillic acids) and flavonoids (rutin, quercetin, kaempferol) (56), and these compounds might be able to interact directly with glucose and fructose intestinal transporters (Figure 3) such as GLUT2, GLUT5, and SGLT1 (57, 58). This constitutes another potential explanation for the inhibitory effect of silverskin extracts on the intestinal absorption of sugar. In reality, the evidence that coffee silverskin extracts, CGA, caffeine, and melanoidins can reduce sugar absorption in the intestinal lumen is still scarce and more studies are needed.

In what concerns to sugar consumption, fructose intake plays a prominent role, and its intestinal absorption and metabolism have been closely correlated with obesity and MetS (27). The bioactive components present in silverskin extracts (fiber, phenolics, flavonoids) were shown to inhibit the absorption of this sugar by intestinal epithelial cells *in vitro* (57). However, more research is still needed to better understand this effect.

### II—Effects on glucose metabolism

The CGA family are specific competitive inhibitors of glucose-6-phosphate translocase enzyme in rat liver microsomes, which is involved in the regulation of blood glucose levels (59), thus exerting a hypoglycaemic activity (34) and modulating glucose metabolism (60).

At the intracellular level, CGA activates adenosine monophosphate-activated protein kinase (AMPK), a sensor and regulator of cellular energy balance, responsible for GLUT2 and GLUT4 translocation from intracellular to plasma membranes (Figure 3), thus increasing glucose blood removal and leading to beneficial metabolic effects, such as the inhibition of fatty acid synthesis and hepatic glucose production, contributing to the regulation of lipid and glucose metabolism (45, 59). Also, CGA presented insulin-sensitizing activity by activating glucose uptake through AMPK pathway in hepatocytes and skeletal muscle cells (60).

On the other hand, in the muscle, caffeine is able to stimulate AMPK (61) and to increase the amount of glucose transporters (GLUT4) (62), improving glucose uptake and insulin sensitivity in the body, and playing an important role in glucose homeostasis (61, 63). Moreover, in adipose cells, caffeine was able to inhibit insulin-induced Akt activation, resulting in decreased glucose transport and GLUT4 translocation (64).

As to silverskin, consumption of silverskin aqueous extract reduces fasting plasma glucose concentrations, increase the sensitivity to insulin, and slow the appearance of glucose in circulation after a glucose load (45, 65).

Likewise, a recent work suggested that aqueous extracts from coffee silverskin (125 μg/mL) stimulated phosphatidylinositol 3-kinase (PI3K) and AMPK signaling (66). The effect on insulin and PI3K pathways improved hepatic cell insulin sensitivity (*in vitro*), and diminished lipogenesis and gluconeogenesis (67). In this regard, ferulic acid, an important metabolite of feruloylquinic acids, has been shown to increase the expression levels of PI3-K and GLUT4, thereby increasing glucose uptake in adipocytes (55).

A recent study, using aqueous extract of coffee silverskin on hepatic cells (10 to 500 μg/mL), suggested that bioactive compounds, particularly CGA, may regulate glucose metabolism and prevent the development of NAFLD by preventing hyperglycemia and systemic insulin resistance (67).

Lastly, according to a recent randomized controlled trial, coffee melanoidins seem to be able to reduce blood glucose peak and insulin response by potentiating the effect of ghrelin and attenuating the response of orexin-A and β-endorphin (41). Therefore, because their basic chemical composition is similar, the melanoidins in the coffee silverskin extract might be expected to have a similar effect.

To summarize, the results of the numerous studies suggest that silverskin has a vast and flexible potential for impact on glucose metabolism, and that CGA and caffeine seem to be the main contributors to these effects of silverskin extract (Figure 3).

### III—Effects on lipid metabolism

Dyslipidemia increases the risk of cardiovascular diseases and, consequently, of morbimortality (68). It is a multifactorial disorder observed in many metabolic disorders such as type II diabetes, obesity and MetS, that includes hepatic overproduction of very low-density lipoproteins (VLDL), decreased triglycerides lipolysis, impaired peripheral free fatty acid (FFA) trapping, increased FFA fluxes from adipocytes to the liver and other tissues (Figure 3), and a higher formation of small dense low-density protein (LDL). In this context, del Castillo et al. (65) have pointed out that silverskin aqueous extract (140 mg/Kg) was able to reduce total cholesterol and triglyceride plasma levels in rats and suggested that one possible mechanism of action was the inhibition of pancreatic lipase, a key enzyme for fat digestion. This liporegulatory character might have preventive and therapeutic effects in obesity and MetS contexts (65). Additionally, a work based on an animal model showed that a silverskin-based beverage had an inhibitory effect on fat accumulation, suggesting it could be a natural alternative for the prevention of obesity and MetS (69) (Figure 3).

Several studies associated the anti-obesity effect of coffee to its bioactive compounds (CGAs, caffeine and melanoidins), which are also present in coffee silverskin, and different mechanisms have been proposed by which they regulate lipid metabolism, including modulation of cell signaling, inhibition of pancreatic lipase, regulation of hepatic lipid metabolism-related enzymes, and reduction in hepatic fat accumulation in the rat model (70). In addition, a meta-analysis by Tabrizi et al. (71) suggested that the long-term consumption of caffeine sources might protect against type II diabetes through increased metabolic rate and thermogenesis, and stimulation of fat oxidation and free fatty acid release from peripheral tissues mediated by AMPK induction (Figure 3).

Using an adipocyte cell line, Rebollo-Hernanz et al. (66) demonstrated that a coffee silverskin aqueous extract (31 μg/mL) was able to inhibit cell differentiation, increase adipocyte lipid metabolism and induce lipolysis through the regulation of lipases. These effects seem to occur mainly through inactivation of ERK, JNK, and NF-κB signaling pathways (66) (Figure 3).

Furthermore, an animal model study with diabetic rats using ferulic acid (a major metabolite of feruloylquinic acids) was able to reduce the level of total cholesterol, low density cholesterol, very low-density lipoprotein, and triglycerides (72).

Recently, a meta-analysis of randomized controlled trials concluded that caffeine has been widely used as a practical approach to obesity control by increasing the release of norepinephrine and dopamine and therefore stimulating neuronal activity in different brain regions, which in turn can decrease body weight and the fat content. Furthermore, this compound can increase fat oxidation by inhibiting phosphodiesterase and suppressing the inhibitory effects of adenosine on noradrenaline release, demonstrating that caffeine intake might promote weight, body mass index and body fat reduction (71).

### IV—Antioxidant effect

As aforementioned, the antioxidant capacity of coffee silverskin is related to the presence of natural constituents like CGA and caffeine, and compounds formed during coffee roasting (melanoidins) (21, 36). Regarding caffeine, some of the reported beneficial health effects are linked to its antioxidant properties (73). A work conducted by del Castillo and colleagues have suggested that caffeine (0.434 mg per dose of aqueous coffee silverskin extract) were able to protect the pancreas against oxidative stress after streptozotocin-induced diabetes in rats (74).

In this context, we should also look at the potential of metabolites of these bioactives. Thus, in a diabetes model, animals treated with a typical CGA metabolite (ferulic acid) showed improved activities of the antioxidant enzymes, SOD and catalase, and reduced glutathione in pancreatic tissue (75).

An *in vitro* study using hepatic cells verified that silverskin aqueous extracts (100 μg/ml) could protect cells from benzo(a)pyrene, an oxidative agent-induced DNA damage. The reduction on DNA strand breaks was suggested to be related to the presence of antioxidant agents such as CGAs and roast-associated constituents (76).

Moreover, melanoidins are known to possess antiglycative, chelating and antioxidant properties (77). Thus, these compounds together with CGA and caffeine may possess a synergic inhibitory effect on the formation of advanced glycation end products (AGEs) and related diseases (78).

Indeed, studies in animals and humans found that the ingestion of phytochemicals from coffee (and by extrapolation, its by-products) induce an adaptive cellular response characterized by upregulation and *de novo* synthesis of enzymes involved in cell defense and repair (79). In particular, the anti-inflammatory and antioxidant features of CGA are well–recognized (80) (Figure 3).

In this context, the nuclear factor erythroid 2-related factor 2 (Nrf2) is an important cellular target for physical binding of coffee phytochemicals (81, 82). This complex is a cellular key regulator in association with AMPK and sirtuins. Melanoidins, CGAs, and their degradation products have been reported as robust activators of Nrf2 activity (82, 83) and this effect may contribute to the possible health effects of coffee (79) and its by-products. The liver appears to be an important site of coffee bioactivity, by improving fat oxidation and thus lowering the risk of steatosis. Furthermore, these phytochemicals are linked to the preservation of functional pancreatic β cell mass *via* improved mitochondrial function and a reduction in endoplasmic reticulum stress (Figure 3). Indeed, long-term preservation of liver and β cell functions may account for the association between regular coffee consumption and a lower risk of type 2 diabetes (79). So, coffee silverskin extract has an interesting bioactive cluster, emphasizing the potential of this by-product as an unconventional functional constituent for the food industry (21, 36).

### V—Interaction with gut microbiota

The colonic microflora plays a crucial role upon the fate of CGA metabolites (caffeic and ferulic acids), by converting them into dihydroferulic acid, which can then be absorbed (84). On the other hand, melanoidins appear to induce metabolic effects like those of dietary fiber from cereals linked to polyphenols (modulation of digestive enzymes, intestinal microbiota and control of oxidative processes in the gut) (41). Also, they are metabolized by the intestinal microbiota, being used as carbon and nitrogen sources by the hindgut microflora, and exerting a prebiotic activity (Figure 3) through the modulation of bacterial colon population and supporting *Bifidobacteria* growth (85).

CS, Coffee silverskin; CGA, Chlorogenic acids; AMPK, 5' adenosine monophosphate-activated protein kinase; FFA, Free fatty acids; GLP1, Glucagon-like peptide-1; GLUTs, Glucose transporters; SGLT1, Sodium/glucose cotransporter 1; GIP, Glucose-dependent insulinotropic polypeptide; G-6Pase, Glucose-6-phosphate translocase; PI3K, Phosphatidylinositol 3-kinases; NRF2, Nuclear factor erythroid 2-related factor 2; AGEs, Advanced glycation end products; SIRT1, Sirtuin 1.

Additionally, melanoidins contribute to the prolongation of satiety, more specifically, their metabolites stimulate the secretion of GLP-1 by the enteroendocrine cells, causing a delay in the digestion of sugars (41). Interestingly enough, a recent study showed that the preventive and therapeutic effect of CGA in relation to obesity may be related to the regulation of gut microbiota (86). Indeed, the gut microbiota is the main driver of both intra- and inter-individual variations in metabolism of dietary bioactive small molecules (47). In this regard, it is important to note that research into melanoidins metabolism by gut microbiota is valuable because these biotransformed metabolites play an important role in the protection of colorectal cells from cancer by reducing inflammation and boosting tumor cell apoptosis. Moreover, data from an animal model study showed that caffeine consumption neutralizes the shift in the obesogenic (Firmicutes/Bacteroides) ratio in gut microbiota, resulting from a western diet (87). So, the ability of main bioactive compounds from coffee silverskin to modulate gut microbiota may play an important role in the prevention/treatment of MetS.

## Conclusions

Coffee silverskin compounds seem to contribute to a potential protective effect against MetS through complementary mechanisms of action: (a) interaction with intestinal sugar transporters, hormones, and signaling pathways acting on glucose metabolism, particularly AMPK; (b) improvement of glucose uptake and insulin sensitivity; (c) lowering total cholesterol and triglyceride plasma levels, possibly through the inhibition of pancreatic lipase; (d) reduction of inflammatory cytokine production in adipose tissue; (e) protection against oxidative DNA damage; and (f) acting as interesting players in the regulation of gut microbiota.

By this way, coffee silverskin appears as a very interesting matrix to be used directly or to develop functional ingredients to enrich foods or to produce dietary supplements with potential beneficial effects against MetS. Simultaneously, the valorization of this by-product that, up to date, is being discarded by the coffee roasting industries, would also respond to the sustainability and circular economy needed in the coffee chain.

Lastly, it is essential to note that coffee silverskin extract is not a competitor to the coffee beverage and can be consumed as a supplement or used as a functional ingredient to enrich beverages and foods. Silverskin valorization has a sustainable footprint and a wealth of bioactive compounds as key factors and can be consumed by a wide range of people, regardless of whether they are coffee drinkers or dislike the taste of coffee.

## Author contributions

Conceptualization, Research/Investigation: NA, JP, and RA. Writing–original draft preparation: NA and JP. Writing–review and editing, Funding acquisition: BO, FM, and RA. Visualization: NA and FM. Supervision: FM and RA. Project administration: RA and BO. All authors contributed to the article and approved the submitted version.

## Funding

The open access publication fee of this paper was funded by AgriFood XXI I&D&I project (NORTE-01-0145-FEDER-000041) cofinanced by European Regional Development Fund (ERDF) through the NORTE 2020 (Programa Operacional Regional do Norte 2014/2020).

## Conflict of interest

The authors declare that the research was conducted in the absence of any commercial or financial relationships that could be construed as a potential conflict of interest.

## Publisher's note

All claims expressed in this article are solely those of the authors and do not necessarily represent those of their affiliated organizations, or those of the publisher, the editors and the reviewers. Any product that may be evaluated in this article, or claim that may be made by its manufacturer, is not guaranteed or endorsed by the publisher.
